# Stability of Imprinting and Differentiation Capacity in Naïve Human Cells Induced by Chemical Inhibition of CDK8 and CDK19

**DOI:** 10.3390/cells10040876

**Published:** 2021-04-12

**Authors:** Raquel Bernad, Cian J. Lynch, Rocio G. Urdinguio, Camille Stephan-Otto Attolini, Mario F. Fraga, Manuel Serrano

**Affiliations:** 1 Cellular Plasticity and Disease Group, Institute for Research in Biomedicine (IRB Barcelona), Barcelona Institute of Science and Technology (BIST), 08028 Barcelona, Spain; raquel.bernad@tum.de (R.B.); cian.lynch@irbbarcelona.org (C.J.L.); 2 Nanomaterials and Nanotechnology Research Center (CINN-CSIC), Cancer Epigenetics and Nanomedicine Laboratory, 33940 El Entrego, Spain; rgurdinguio@gmail.com (R.G.U.); mffraga@cinn.es (M.F.F.); 3 Health Research Institute of Asturias (ISPA), 33011 Oviedo, Spain; 4 Institute of Oncology of Asturias (IUOPA), University of Oviedo, 33006 Oviedo, Spain; 5 Rare Diseases CIBER (CIBERER), 33011 Oviedo, Spain; 6 Biostatistics and Bioinformatics Unit, Institute for Research in Biomedicine (IRB Barcelona), Barcelona Institute of Science and Technology (BIST), 08028 Barcelona, Spain; camille.stephan@irbbarcelona.org; 7 Catalan Institution for Research and Advanced Studies (ICREA), 08010 Barcelona, Spain

**Keywords:** pluripotency, naïve, stem, imprinting, primordial germ cells, trophoblast, CDK8, Mediator, enhancers

## Abstract

Pluripotent stem cells can be stabilized in vitro at different developmental states by the use of specific chemicals and soluble factors. The naïve and primed states are the best characterized pluripotency states. Naïve pluripotent stem cells (PSCs) correspond to the early pre-implantation blastocyst and, in mice, constitute the optimal starting state for subsequent developmental applications. However, the stabilization of human naïve PSCs remains challenging because, after short-term culture, most current methods result in karyotypic abnormalities, aberrant DNA methylation patterns, loss of imprinting and severely compromised developmental potency. We have recently developed a novel method to induce and stabilize naïve human PSCs that consists in the simple addition of a chemical inhibitor for the closely related CDK8 and CDK19 kinases (CDK8/19i). Long-term cultured CDK8/19i-naïve human PSCs preserve their normal karyotype and do not show widespread DNA demethylation. Here, we investigate the long-term stability of allele-specific methylation at imprinted loci and the differentiation potency of CDK8/19i-naïve human PSCs. We report that long-term cultured CDK8/19i-naïve human PSCs retain the imprinting profile of their parental primed cells, and imprints are further retained upon differentiation in the context of teratoma formation. We have also tested the capacity of long-term cultured CDK8/19i-naïve human PSCs to differentiate into primordial germ cell (PGC)-like cells (PGCLCs) and trophoblast stem cells (TSCs), two cell types that are accessible from the naïve state. Interestingly, long-term cultured CDK8/19i-naïve human PSCs differentiated into PGCLCs with a similar efficiency to their primed counterparts. Also, long-term cultured CDK8/19i-naïve human PSCs were able to differentiate into TSCs, a transition that was not possible for primed PSCs. We conclude that inhibition of CDK8/19 stabilizes human PSCs in a functional naïve state that preserves imprinting and potency over long-term culture.

## 1. Introduction

Mammalian pluripotency spans a continuum of interconvertible states, each with a distinct set of molecular and functional attributes. Naïve pluripotency is functionally and transcriptionally comparable to the cells of the pre-implantation epiblast [1,2], while primed pluripotency resembles post-implantation epiblast cells [3]. Mouse naïve pluripotency is efficiently induced and stabilized in vitro by adding two kinase inhibitors for MEK and GSK3 (abbreviated 2i) to the culture medium [4]. Building on the success of 2i to stabilize naïve mouse pluripotent stem cells (PSCs), several groups have reported chemical cocktails that include 2i and that also induce naïve-features in human PSCs [5,6,7,8,9,10,11]. Remarkably, while all 2i-based cocktails for the induction of naïve human pluripotency display an improvement in culture homogeneity, long-term expansion of human PSCs in 2i-based naïve media results in karyotypic instability, global genomic DNA demethylation, gradual loss of imprinting, and deficits in developmental potency [12,13,14,15,16]. These detrimental effects have been attributed to the chronic inhibition of MEK, a kinase whose multiple functions include the maintenance of DNA methylation by DNMT1 [17,18]. In support of this, reduction of the amount of MEK inhibitor partially alleviates the genomic instability of 2i-based naïve human PSCs [19].

Recently, we reported that the naïve state can be induced and stabilized by stimulating the Mediator complex at enhancers through the inhibition of its kinase repression module, composed of the highly similar kinases CDK8 and CDK19 [20,21]. We showed that the simple addition of one chemical, a CDK8 and CDK19 inhibitor (CDK8/19i), is sufficient to induce the primed to naïve transition in mouse and human PSCs. Interestingly, the inhibition of CDK8 and CDK19 does not deplete genomic DNA methylation and, thereby, does not result in karyotypic abnormalities in human PSCs [20]. Moreover, CDK8/19i-naïve human PSCs efficiently form embryoid bodies and teratomas in mice [20], a property that is generally compromised in 2i-naïve human PSCs [12].

Here, we assess the stability of genomic imprints after long-term culture of human PSCs in the presence of CDK8/19i, and the capacity of CDK8/19i-treated human PSCs to differentiate into primordial germ cell (PGC)-like cells (PGCLCs) and trophoblast stem cells (TSCs).

## 2. Materials and Methods

### 2.1. Human PSC Resources

Human cells were obtained from commercial sources or from other academic institutions. The use of the human pluripotent stem cells employed in this study was approved by the Spanish National Advisory Committee for Human Tissue and Cell Donation and Use and by the Catalan Department of Health (project numbers: 0336S/2473/2017; 0336/747/2018). WIBR3 and OCT4-∆PE ESCs were obtained from the laboratory of Jacob Hanna (Weizmann Institute of Science, Rehovot, Israel). H1 ESCs, and CB5, D2#2, and D2#4 human iPSCs were obtained from the laboratory of Nuria Montserrat (IBEC, Institute for Bioengineering, Barcelona, Spain). H1 ESCs were bought and used in agreement with WiCell.

### 2.2. Human PSC Culture Conditions

Human PSCs (hPSCs) were maintained in conventional primed conditions by culture on growth factor-reduced phenol red-free Matrigel (Corning #356231, Corning, NY, USA) or human ESC-qualified Matrigel (Corning #354277) with mTeSR1 media (Stem Cell Technologies). Cultures were passaged every 5–7 days manually using 0.5 μM EDTA/1xPBS (Gibco, Grand Island, NY, USA). To reset hPSCs to CDK8/19i-naïve state, cells were maintained on Matrigel (Corning #356231 or #354277) using mTeSR1 (Stem Cell Technologies, Vancouver, Canada); the basal media was supplemented with 20 ng/mL of rhLIF (Peprotech, Rocky Hill, NJ, USA) plus 0.4 μM of CNIO-CDK8/19 inhibitor (ETP-47799), as reported [20]. Differentiation of OCT4-∆PE hPSCs as control for the FACS experiments was induced with 10 μM retinoic acid (RA, Sigma, St. Louis, MO, USA) for 5 days [7].

### 2.3. Derivation of Primordial Germ Cells (PGCs)

For short-term pre-exposure, hPSCs were exposed for 4 days to naïve conditions. Naïve cocktails used were: CDK8/19i [0.4 μM of CNIO-CDK8/19 inhibitor and 20 ng/mL hrLIF (Peprotech)], as reported [20]; or the 2i-based PXGL [1 μM PD0325901 (Axon Medchem, Reston, VA, USA), 2 μM Gö6983 (Selleckchem, Houston, TX, USA), 2 μM XAV939 (Selleckchem) and 20 ng/mL hrLIF (Peprotech)] [8]. PGCs-like derivation was performed as previously reported [22]. Briefly, hPSCs were induced to iMeLCs (induced mesoderm-like cells) and then into PGC-like cells. For the induction of iMeLCs, hiPSCs were plated at a density of 5 × 10^4^ cells/cm^2^ onto a fibronectin-coated (Millipore, FC010) 6-well plate in the GK15 medium [GMEM (Thermo Fisher Scientific, Waltham, MA, USA) with 15% KSR (Gibco), 0.1 mM NEAA (Gibco), 2 mM L-glutamine (Gibco), 1 mM sodium pyruvate (Gibco), and 0.1 mM 2-mercaptoethanol (Gibco)] supplemented with 50 ng/mL activin A (R&D, Minneapolis, MN, USA), 3 μM CHIR99021 (Axon Medchem,) and 10 μM Y-27632 (Selleckchem). After 48 h, cells (iMeLCs) were dissociated into single cells with TrypLE Select (Thermo Fisher Scientific) and were aggregated in a low-cell-binding V-bottom 96-well plate (Thermo Fisher Scientific, 81100574) at 3000 cells per well in the GK15 medium supplemented with 200 ng/mL BMP4 (R&D Systems), 100 ng/mL SCF (R&D Systems), 50 ng/mL EGF (R&D Systems), 20 ng/mL hrLIF (Peprotech), and 10 μM Y-27632 to be induced into hPGCLCs.

### 2.4. Derivation of Trophoblast Stem Cells (TSCs)

For short-term pre-exposure, hPSCs were exposed for 4 days to naïve conditions. Naïve cocktails used were: CDK8/19i cocktail [0.4 μM of CNIO-CDK8/19 inhibitor and 20 ng/mL hrLIF (Peprotech)], as reported [20], or the 2i-based cocktail PXGL [1 μM PD0325901 (Axon Medchem), 2 μM Gö6983 (Selleckchem), 2 μM XAV939 (Selleckchem) and 20 ng/mL hrLIF (Peprotech)] [8]. TSCs derivation was performed as previously reported by Kojima and colleagues [23]. Naïve and primed hPSCs were single-cell dissociated by TrypLE Express, and 0.5 × 10^6^ cells were seeded in a 6-well plated pre-coated with 5 mg/mL Collagen IV (#354233, Corning) and cultured in TS medium [DMEM/F12 (D6421, Sigma), 0.1 mM 2-mercaptoethanol (Gibco), 0.2% FBS (Gibco), 0.3% BSA (Sigma), 1% ITS-X (Gibco), 1.5 μg/mL L-ascorbic acid (Sigma), 50 ng/mL EGF (PeproTech), 2 μM CHIR99021 (Axon Medchem), 0.5 μM A83-01 (Tocris, Bristol, UK), 1 μM SB431542 (PeproTech), 0.8 mM VPA (Sigma) and 5 μM Y-27632 (Selleckchem)] [24]. Cells were passaged every 5–7 days in single cells using TrypLE Express on pre-coated Collagen IV plates.

### 2.5. Teratoma Formation Assay

Mice were housed at the specific pathogen-free (SPF) barrier area of the Institute for Research in Biomedicine (IRB Barcelona) in Barcelona. Two injections of human H1 and D2#2 PSCs were performed per mouse in testes of SCID beige mice (8 weeks of age, ENVIGO). 2 × 10^6^ cells in 30 μL of mTeSR media were injected per testis. Teratomas were extracted when reaching a size of about 1 cm.

### 2.6. RNA Isolation and qRT-PCR

Total RNA from cells was extracted on column by RNeasy kit with DNA digestion following the provider’s recommendations (Qiagen #74104, #79254, Hilden, Germany) or with Trizol (Invitrogen, Waltham, MA, USA) according to the manufacturer’s recommendations. Up to 1 μg of total RNA was retro-transcribed into cDNA using iScriptTM cDNA Synthesis kit (BioRad #170-8891, Hercules, CA, USA) following the manufacturer’s protocol. Quantitative real-time–PCR (qPCR) was performed using GoTaq^®^ qPCR Master Mix (Promega A6002) in a QuantStudio 6 Flex thermocycler (Applied Biosystem). Input normalization of all the quantitative real-time–PCR (qRT–PCR) data was by the ∆∆Ct method using the housekeeping gene GAPDH as indicated in each figure. Primer sequences used for mRNA analyses are listed in Table S1.

### 2.7. RNA-Seq Transcriptomic Analyses

Samples of total RNA from long-term cultured (>15 passages) human PSCs (D2#2, D2#4, H1, CB5 and WIBR3) in the absence or presence of CDK8/19i. RNA preparations with RIN numbers in the range of 9.0 to 10 (Agilent 2100 Bioanalyzer, Santa Clara, CA, USA) were used. For library construction 10 ng of total RNA samples were processed with the SMART-Seq v4 Ultra Low Input RNA Kit (Clontech, Mountain View, CA, USA), following the manufacturer’s instructions. Resulting cDNA was sheared on a S220 Focused-ultrasonicator (Covaris, Woburn, MA, USA) and subsequently processed with the “NEBNext Ultra II DNA Library Prep Kit for Illumina” (NEB #E7645). Briefly, oligo(dT)-primed reverse transcription was performed in presence of a template switching oligonucleotide, double-stranded cDNA was produced by 11 cycles of PCR and submitted to acoustic shearing. Fragments were processed through subsequent enzymatic treatments of end-repair, dA-tailing, and ligation to Illumina adapters. Adapter-ligated libraries were completed by limited-cycle PCR (8 cycles). The resulting directional cDNA libraries were sequenced for 50 bases in a single-read format, instrument: Illumina HiSeq2500 Primary data processing: Image analysis, per-cycle base-calling and quality score assignment was performed with Illumina Real Time Analysis software. Conversion of Illumina BCL files to bam format was performed with the Illumina2bam tool (Wellcome Trust Sanger Institute—NPG). The complete set of reads has been deposited in the GEO repository (accession numbers: GSE127186 and GSE152378). Paired-end reads were aligned to the hg19 human genome using STAR [25] with default parameters. Gene counts were computed using the Rsubread package [26] with the inbuilt annotation for the hg19 genome. Differential expression was performed using DESeq2 [27] using the cell line as covariate. Genes were sorted by the shrunk fold change as computed by the “lfcShrink” function.

### 2.8. Differential Gene Expression Comparison of Published Studies

Gene set enrichment analysis was performed using the pre-ranked GSEA function as implemented by the Broad Institute [28]. First, we generated two gene signatures of differentially expressed genes (DEGs), either up-regulated or down-regulated, in short-term cultured human PSCs (D2#2, D2#4, HERVH and H1) (raw data reported in [20] and accessible at GSE127186). We defined gene signatures as DEGs with adjusted FDR q-value lower than 0.1 and log2 fold changes larger than 1 (for up-regulated genes) or lower than −0.5 (for down-regulated genes). These short-term up and down signatures for CDK8/19i-naïve cells are in Table S2. These signatures were used to perform GSEA against a ranked list for DEGs in the long-term cultured PSCs in the absence or presence of CDK8/19i (*n* = 5 cell lines, see above RNA-seq and Table S3). As a control for other naïve cells obtained with 2i-based cocktails, we used ranked lists of DEGs from [6,7,9,10,15,29,30], also detailed in [20]. For the data generated in this study, genes were ranked by the shrunk fold change as computed by the “lfcShrink” function. Gene set enrichment was run with standard GSEA settings. Results were summarized through the Normalized Enrichment Score (NES). Data with *p* < 0.05 and FDR *q* < 0.05 were considered significant and marked with an asterisk (*) in the heatmap of GSEA NES scores: * *p* < 0.05, ** *p* < 0.01, *** *p* < 0.001. Heatmaps were plotted using the ggplot2 package [31] in R [32].

### 2.9. DNA Methylation

DNA methylation analyses were performed by the laboratory of Mario Fraga (CINN, Oviedo, Spain). DNA was purified and CpG methylation status at individual CpG sites of DMR (Differentially Methylated Regions) regions, at imprinted genes, was assessed by DNA bisulphite-conversion and pyrosequencing, as described [33]. DNA was purified following the standard phenol:chloroform:isoamyl alcohol (25:24:1) (Sigma) extraction protocol. Bisulfite modification of DNA was performed with the EZ DNA Methylation-Gold kit (#D5006, Zymo Research, Irvine, CA, USA) following the manufacturer’s instructions. The set of primers for PCR amplification and sequencing were designed using the specific software Pyrosequencing Assay Design (version 2.0.01.15) from Biotage AB. Primer sequences were designed to hybridize with CpG free sites to ensure methylation-independent amplification, one containing a Biotin mark (Btn) at the 5′ end (Table S1). After PCR amplification and purification (based on the biotin mark) of the region of interest with the specific primers, pyrosequencing was performed using PyroMark Q24 reagents, Vacuum Prep Workstation (Biotage AB, Uppsala, Sweden) and specific-designed sequencing primer (Table S1). Sequencing primers were designed covering 4 or 5 CpGs per DMR. Results were analysed with PyroMark Q24 2.0.6 (Qiagen) software and equipment, obtaining percentage of methylation for each specific CpG site studied.

### 2.10. Exome Sequencing

Genomic DNA (gDNA) was purified following the manufacturer’s instructions (Qiagen #69504). gDNA samples were purified using AMPure DNA Magnetic Beads and a RNASe treatment was performed on the samples. The purified gDNA was quantified using DNA Hs Qubit Assay and fragmented in 250–300 bp size using COVARIS System. Exome capture hybridization was performed according to the manufacturer’s instructions (AGILENT SureSelect XT Kit, Santa Clara, CA, USA), followed by Exome capture Library preparation (NEBNext^®^ Ultra™ DNA Library Prep Kit for Illumina, Ipswich, MA, USA). Next-generation sequencing was performed at HiSeq 2500 Sequencing System Illumina, Sequencing Type 125 nt Pair End. The Picard suite (Picard tools http://broadinstitute.github.io/picard/; accessed on 30 September 2020) was used to trim Illumina adapter. Paired-end reads were aligned to the hg19 human genome using bwa [34] followed by removal of duplicate reads using sambamba [35]. Following the best practices recommended by the authors of GATK [36], read quality was recalibrated (GATK version 4.0.6.0). GATK’s Haplotype caller [37] was used to discover single nucleotide and short polymorphisms. SNPs were filtered using the following thresholds: FS > 60.0, QD < 2.0, MQRankSum < −12.5, ReadPosRankSum < −8.0 and MQ < 40.0.

### 2.11. Imprinting Score

A list of putative imprinted genes was compiled from [12,14,38] and references therein. For each gene, all SNPs passing filters were selected. For RNAseq samples corresponding to each Exome seq sample, reads aligning to selected SNPs were counted and the percentage of the alternative allele was computed. We filtered out positions with less than 10 reads. Following the thresholds in [38], we defined three categories of expression depending on the alternative allele fraction: Monoallelic 0 < AAF ≤ 0.14; Partially-monoallelic 0.15 < AFF ≤ 0.29; Biallelic 0.3 < AFF.

### 2.12. Statistical Analysis

For differential gene expression by RNAseq, a threshold of FDR q-value of *q* < 0.05, or *q* < 0.01 was applied, as indicated in each case. In GSEA analysis, the standard threshold for significance was applied, where *p* < 0.05 and FDR *q*-value < 0.05.

## 3. Results

### 3.1. CDK8/19i Stabilizes Naïve Human PSCs over Long-Term Passaging 

**Figure 1:**
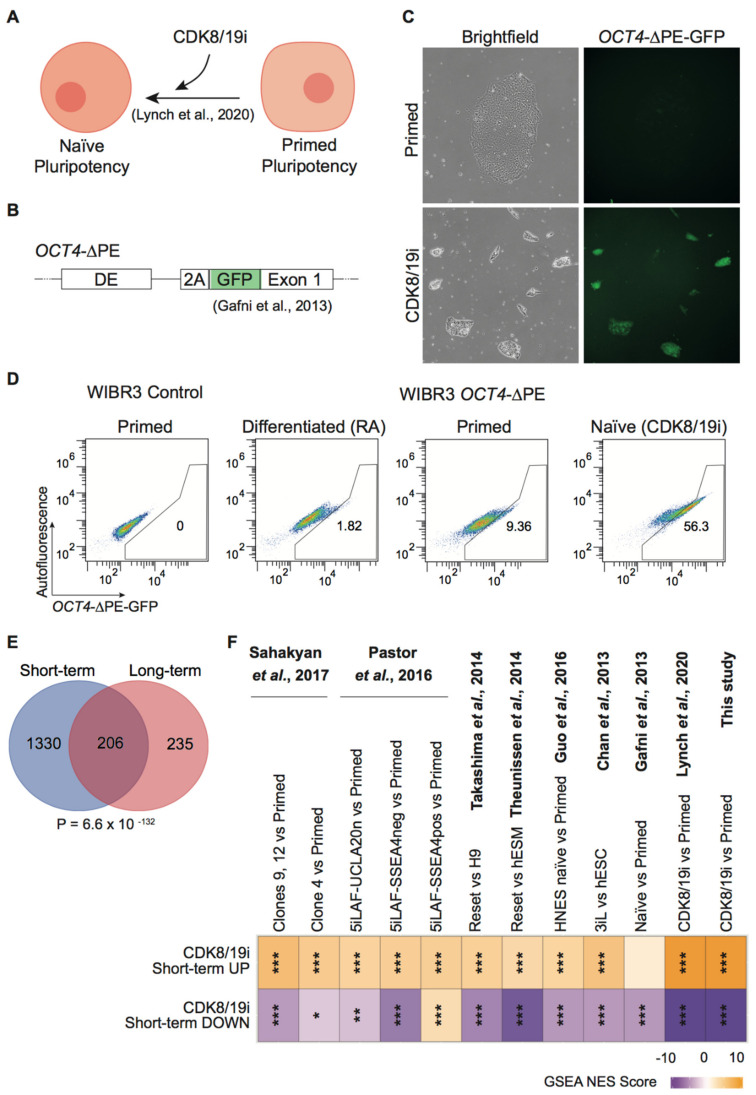
Stability of hPSC-naïve pluripotency after long-term culture with CDK8/19i. (**A**) Experimental scheme for inducing naïve conditions using the previously reported CDK8/19i chemical approach [20]. (**B**) Schematic diagram of *OCT4*-∆PE-GFP reporter [7] for naïve human pluripotency based on the *OCT4* distal enhancer (DE). (**C**) Brightfield images showing colony morphology (left panels) or *OCT4*-∆PE-GFP expression (right panels), both in primed (upper images) or CDK8/19i-treated (bottom images) WIBR3 reporter hESCs. (**D**) Comparative fluorescent cytometry analyses of WIBR3 reporter hESCs after differentiation with retinoic acid (RA), primed conditions, or CDK8/19i-naïve conditions, as well as the parental line WIBR3 (primed) as negative control (left panel). Numbers indicate the % of cells in the GFP-positive gate. (**E**) Overlap and hypergeometric significance of differentially expressed mRNAs in short- and long-term CDK8/19i naïve (relative to primed). (**F**) Heatmap of the normalized enrichment scores (NES) of the gene-set enrichment analyses (GSEA). As signatures, we used the differentially up- or down-regulated genes in short-term CDK8/19i-naïve (relative to primed). These signatures were tested on the ranked lists of gene expression changes in previously published naïve conditions (see references) and in our long-term adapted CDK8/19i hPSCs (this study). For the last comparison, we used a total of 5 hPSCs: D2#2, D2#4, H1, CB5 and WIBR3. Statistical significance of GSEA NES scores in the heatmaps is indicated using the symbol “*”. FDR *q* < 0.05; * *p* <0.05, ** *p* < 0.01, *** *p* < 0.001.

We have previously reported that human PSCs cultured in the presence of a CDK8/19 inhibitor for about 5 to 10 passages phenocopy molecular features of naïve pluripotency (Figure 1A) at the transcriptional, proteome, phospho-proteome and metabolome levels [20,21]. Remarkably, all media cocktails based on 2i for the induction of human naïve pluripotency result in karyotypic instability, gradual erasure of DNA methylation on imprinted genes and deficits in developmental potency [12,13,14,15,16]. Published literature considers stable long-term culture as those cultures that exceed 10 passages, and this is the time required for the observation of karyotypic abnormalities [12,19]. Fewer passages may not be enough to fully manifest this problem. We wondered if the CDK8/19i-naïve state can be preserved for long periods of culture. For this, we used a reporter of the naïve state that we had not tested previously, in particular, a reporter based on the *OCT4* (also known as *POU5F1*) enhancer (Figure 1B). It is well described that *OCT4* expression is primarily dependent on the proximal enhancer (PE) in primed human PSCs, while naïve human PSCs are characterized by the predominant utilization of the distal enhancer (DE) [7,10]. After 15 passages (~90 days in culture) in the presence of CDK8/19i, human WIBR3 PSCs carrying the *OCT4*-∆PE-GFP reporter presented a substantial increase in GFP fluorescence compared to the same cells in the absence of CDK8/19i (Figure 1C). Flow cytometry analyses confirmed a substantial activation of *OCT4*-∆PE-GFP in CDK8/19i-cultured cells compared to their counterparts in the absence of inhibitor (primed conditions), and compared to additional negative controls consisting of retinoic acid-induced (RA) differentiated cells and primed PSCs not carrying the reporter (Figure 1D).

To further support the stability of the naïve state in the presence of CDK8/19i, we performed whole-transcriptome profiling on long-term (>10 passages) adapted CDK8/19i-naïve and primed human PSCs from 5 different cell lines, namely, 3 iPSCs (CB5, D2#2, D2#4) and 2 ESCs (WIBR3, and H1) (see Methods for accession number in public databases). First, we observed a significant overlap between the differentially expressed genes in short-term and long-term CDK8/19i cultures (both relative to primed cells) (Figure 1E; see Table S4). We also asked if signatures for up-regulated and down-regulated genes in short-term cultured CDK8/19i-naïve (relative to primed cells) (reported in [20]; see Table S2) were enriched in the differentially expressed genes of our newly generated transcriptomes of long-term CDK8/19i cultured cells (*n* = 5) (see Table S3). Importantly, the short-term CDK8/19i naïve signatures were highly enriched in the long-term CDK8/19i-naïve cells (Figure 1F). The short-term CDK8/19i-naïve signatures were also compared with previously published 2i-based naïve data sets [6,7,9,10,15,29,30] and in all cases showed high enrichment scores similar to the ones obtained with our long-term CDK8/19i-naïve cells (Figure 1F). Therefore, we conclude that the naïve state achieved by inhibition of the Mediator kinases CDK8/19 is stable over long-term culture.

### 3.2. Long-Term Preservation of Genomic Imprints in CDK8/19i-Naïve Human PSCs

Uncontrolled global DNA demethylation and loss of imprints have been reported for all tested 2i-based naïve human PSCs [12,13,14,15,16]. Recent studies have shown that the inhibition of MEK signaling is directly responsible for these undesirable effects [19,39]. Considering that CDK8/19 inhibition does not affect MEK signaling [20], we hypothesized that long-term culture of human PSCs with CDK8/19i might preserve genomic imprinting.

**Figure 2:**
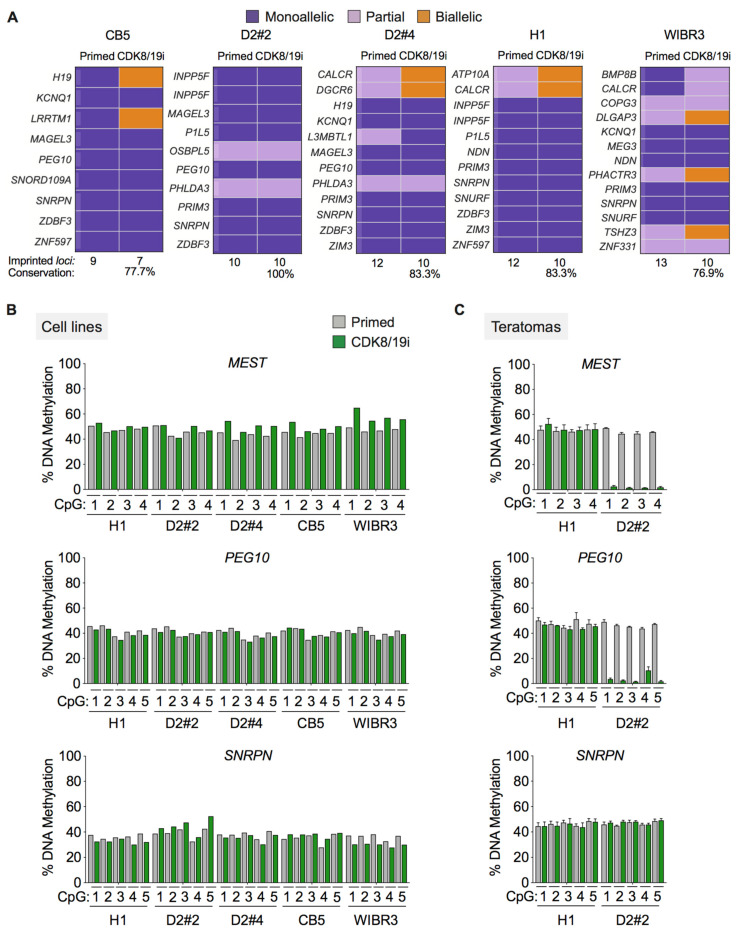
Long-term cultured CDK8/19i-naïve hPSCs retain monoallelic expression and hemi-methylation at imprinted *loci*. (**A**) Heatmaps of allele specific expression data for the indicated informative imprinted genes in primed versus CDK8/19i long-term cultured hPSCs. The lower part of each panel indicates the number and percentage of genes expressed in a monoallelic or partially monoallelic manner after long-term culture in the presence of CDK8/19i. (**B**) CpG methylation status of differentially methylated regions (DMRs) at the indicated imprinted *loci* in five hPSC lines adapted to CDK8/19i for >10 passages. For each DMR, a total of 4 or 5 individual CpGs were analyzed by pyrosequencing. (**C**) CpG methylation status of DMRs in teratomas obtained from primed hPSCs or long-term adapted to CDK8/19i.

First, we followed a strategy previously used based on the combination of exome and RNA sequencing [14,38]. In particular, we performed exome sequencing of 5 human PSCs long-term cultured (>15 passages) in the presence of CDK8/19i, and this was used to identify SNPs in potentially imprinted genes (obtained from [14] and references therein). Then, RNA-seq data were used to discriminate the levels of expression of each allele. Following previous criteria [38], sites displaying 0–14% of minor allele contribution to total gene expression were defined as monoallelic, 15–29% as partially monoallelic and 30–50% as biallelic. Only a small subset (from 9 to 13) of the potentially imprinted genes were informative (presence of SNPs and >10 reads, see Methods) and were expressed in a monoallelic or partially monoallelic manner in human primed PSCs. Interestingly, most (77% to 100%) of the primed monoallelic genes remained monoallelically expressed in long-term cultured CDK8/19i-naïve human PSCs (Figure 2A). This includes the *H19/IGF2* locus, which is known to be particularly susceptible to loss of monoallelic expression in PSCs [14,15,38].

We next assessed by pyrosequencing the methylation status of specific CpG positions within the differentially methylated regions (DMRs) of some maternal (*SNRPN*) and paternal (*MEST*, *PEG10*) imprinted loci. As expected, all the CpG positions analyzed (4 or 5 positions per DMR) were hemi-methylated in primed PSCs (Figure 2B). Interestingly, hemi-methylation was preserved in all the long-term cultured CDK8/19i-naïve human PSCs (Figure 2B). As a further challenge to the stability of the imprints, we injected the long-term cultured human PSCs, both primed and naïve, into SCID mice until the formation of teratomas. Of note, we have previously shown that long-term cultured CDK8/19i-naïve hPSCs are capable of efficient tri-lineage teratoma formation [20]. Genomic DNA from the teratomas was analyzed as before to determine the methylation status of DMR. The teratomas obtained with CDK8/19i-naïve H1 cells preserved hemi-methylation at all the sites analyzed (Figure 2C). In the case of CDK8/19i-naïve D2#2 cells, two DMRs lost their imprints (*MEST* and *PEG10*) and one DMR preserved hemi-methylation (SNRP) (Figure 2C).

Altogether, we conclude that prolonged culture of PSCs in the presence of CDK8/19i does not erase DNA methylation imprints. However, we detected some loss of imprinting after teratoma formation. The high stability of imprinting in CDK8/19i-naïve human PSCs is in contrast with the recent findings that >70% of imprinted DMRs were erased in 2i-based naïve human PSCs [16].

### 3.3. Differentiation Potential of CDK8/19i-Naïve Human PSCs into Primordial Germ Cells

**Figure 3:**
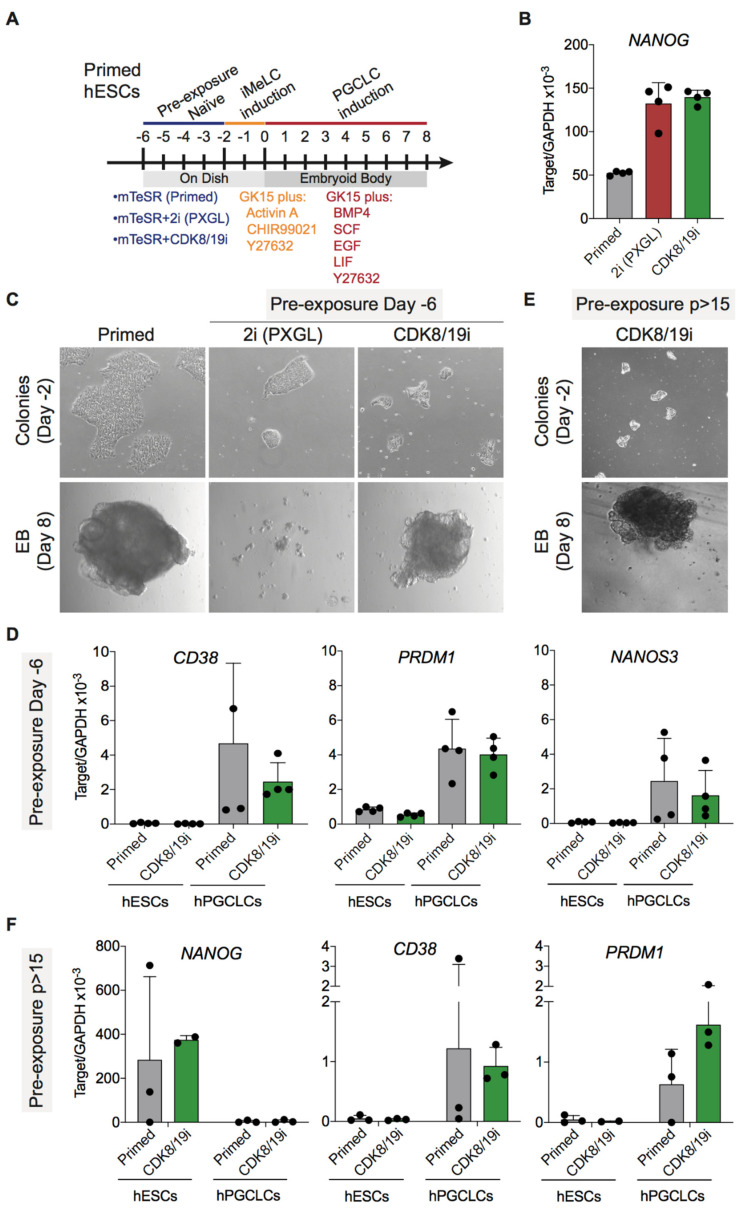
CDK8/19i-naïve hPSCs can differentiate into primordial germ cell-like cells (hPGCLCs). (**A**) Experimental scheme for assessing PGC differentiation of primed, 2i (PXGL) and CDK8/19i-naïve hESCs. iMeLCs: induced mesoderm-like cells. (**B**) mRNA expression level of *NANOG* assessed by qRT-PCR. Up-regulation of *NANOG* is a feature of the naïve state. Data represent mean ± Std Dev from 4 technical replicates. (**C**) Brightfield images showing H1 hESCs before and after exposure to naïve conditions and embryoid bodies (EBs) in hPGCLCs induction conditions at day 8. (**D**) mRNA expression level of selected hPGCLC markers in hESCs and EBs differentiated into hPGCLCs assessed by qRT-PCR. Data of hESC are mean ± Std Dev from 4 technical replicates and data of hPGCLCs are mean ± Std Dev from 4 biological replicates. (**E**) Brightfield images showing H1 hESCs cultured in the presence of CDK8/19i for more than 15 passages (*p* > 15). Lower panel shows EB formation under PGCLC differentiation conditions at day 8. (**F**) mRNA expression level of pluripotency marker *NANOG* and selected hPGCLC markers in H1 cells primed or long-term cultured in CDK8/19i (more than 15 passages) before or after differentiation into hPGCLCs assessed by qRT-PCR. Data are mean ± Std Dev from 3 biological replicates.

As a first test of the differentiation potential of CDK8/19i-naïve hPSCs, we examined their capacity to generate primordial germ cell (PGC)-like cells (PGCLCs). PGCLCs have been recently generated from human PSCs [22,40]. Here, we have employed a two-step protocol that involves supplements to induce mesoderm-like cells (iMeLC induction) followed by formation of embryoid bodies (EBs) in the presence of a specific PGCLC medium (PGCLC induction) [22] (Figure 3A). These studies have shown robust production of hPGCLCs both from primed PSCs or from PSCs briefly pre-treated for 4 days with a 2i-based cocktail known as NHSM [7].

Based on the above reports, we compared first the effect of short pre-treatments with CDK8/19i or with another 2i-based cocktail known as PXGL [8] (Figure 3A). These naïve pre-treatments for 4 days were sufficient to increase the mRNA levels of *NANOG* in primed H1 ESCs, thereby indicating the induction of naïve features by both methods (Figure 3B). After the short naïve pre-treatment, PGCLCs were induced according to the protocol of Kojima et al. [22] (Figure 3A). Notably, primed and CDK8/19i-treated H1 ESCs efficiently formed EBs under PGCLC differentiation conditions (Figure 3C) and up-regulated specific PGCLCs markers, such as *CD38*, *PRDM1*, and *NANOS3* (Figure 3D). PXGL pre-treatment was detrimental for the formation of EBs under PGCLC differentiation conditions (Figure 3C), leading to cell death and preventing the assessment of PGCLC markers. These results indicate that short treatment of primed PSCs with CDK8/19i does not impair their differentiation potential into PGC-like cells.

In a subsequent set of experiments, human H1 cells that had been maintained in the presence of CDK8/19i for more than 15 passages (*p* > 15) were subjected to the above-mentioned PGCLC differentiation protocol. We were not able to obtain long-term cultures of H1 cells in PXGL medium. Interestingly, long-term CDK8/19i-naïve cells were not impaired in the formation of EBs under PGCLC differentiation conditions (Figure 3E) and upregulated PGCLCs markers (*CD38* and *PRDM1*) while downregulating the pluripotency marker *NANOG* (Figure 3F). We conclude that extended treatment with CDK8/19i is not detrimental for the differentiation capacity of PSCs into primordial germ cells.

### 3.4. Differentiation Potential of CDK8/19i-Naïve Human PSCs into Trophoblast Stem Cells

**Figure 4:**
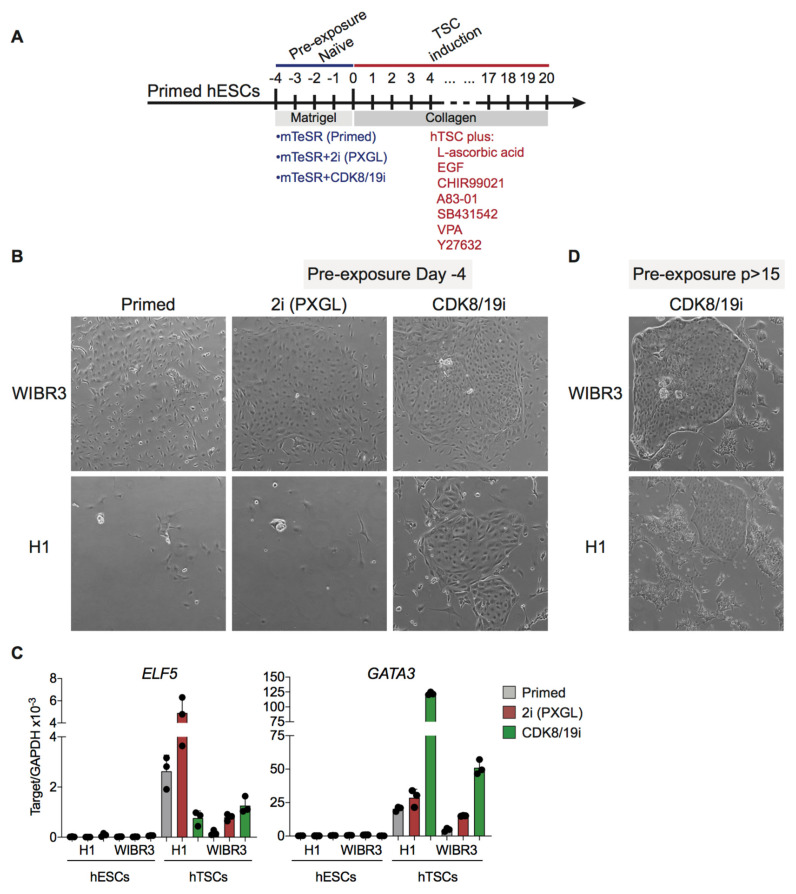
CDK8/19i-naïve hPSCs can differentiate into trophoblast stem cells (hTSCs). (**A**) Experimental scheme for assessing hTSCs differentiation of primed, 2i (PXGL) and CDK8/19i-naïve hESCs. (**B**) Brightfield images showing hTSC-like colonies derived from hESCs (WIBR3 and H1) after exposure to short-term naïve conditions. (**C**) mRNA expression level of selected trophoblast markers assessed by qRT-PCR. Data are mean ± Std Dev of 3 technical replicates. (**D**) Brightfield images showing hTSC-like colonies derived from hESCs (WIBR3 and H1) after more than 15 passages (*p* > 15).

Previous studies have reported that naïve human PSCs can acquire extra-embryonic fates, and this is a distinctive property of the naïve state absent in primed PSCs [16,23]. Here, following the same strategy used above to induce PGCLC differentiation, primed H1 and WIBR3 hESCs were briefly pre-treated for 4 days with CDK8/19i or with 2i-based PXGL to induce naïve features (see above Figure 3B). Cells were then cultured for 20 days in human TSC medium, as previously reported [24] (Figure 4A). In the case of WIBR3 cells, briefly-treated PXGL-naïve and CDK8/19i-naïve cells formed colonies with a typical human TSC morphology (Figure 4B). As expected, primed WIBR3 not exposed to naïve cocktails did not form colonies with TSC morphology (Figure 4B). In the case of H1 cells, TSC colonies were only observed in cells briefly-treated with CDK8/19i, but not in cells briefly-treated with PXGL for 4 days (Figure 4B). The expression of trophoblast marker *GATA3* correlated with the formation of TSC colonies, thus being maximally expressed in cultures derived from CDK8/19i-naïve H1 and WIBR3 cells (Figure 4C). In the case of trophoblast marker *ELF5*, expression was higher under TSC culture conditions but their levels did not correlate with TSC colony formation (Figure 4C).

Finally, cells that had been maintained in the presence of CDK8/19i for more than 15 passages (*p* > 15) were also subjected to TSC induction. As mentioned above for PGCLC differentiation, we were not able to obtain long-term cultures of H1 or WIBR cells in PXGL medium. Interestingly, long-term CDK8/19i-naïve WIBR3 and H1 cells formed colonies with distinct features of trophoblast stem cells (Figure 4D). Together, we conclude that treatment with CDK8/19i, either short term or long term, allows hPSCs to acquire trophoblast stem cell identity.

## 4. Discussion

This study reports three main findings regarding the ability of CDK8/19i to induce naïve features in human PSCs. First, long-term culture (>15 passages) in the presence of CDK8/19i maintains the expression of naïve features, as indicated by the OCT4-∆PE-GFP reporter or global transcriptome analysis. Second, long-term CDK8/19i-naïve hPSCs preserve their genomic imprints intact, including hemi-methylation of differentially methylated regions and monoallelic expression. Third, the naïve state induced by CDK8/19i in hPSCs allows subsequent differentiation into primordial germ cells or into trophoblast stem cells. Importantly, trophoblast differentiation capability is considered characteristic of naïve hPSCs, but not primed hPSCs.

Naïve hPSCs have been previously achieved using a variety of chemical cocktails [12], all of them based on the MEK and GK3 two inhibitors cocktail (2i) initially developed for mouse ESCs [4]. However, 2i-based-naïve hPSCs are, in general, unstable compared to primed hPSCs. This instability includes karyotypic abnormalities and loss of imprinted marks [10,12,15,30]. The inhibition of MEK, a key feature of all the 2i-based cocktails, is considered the key inducer of this genomic instability. In support of this, reduction of the amount of MEK inhibitor in naïve cocktails reduces the accumulation of karyotypic abnormalities [19]. The reason why MEK inhibition results in genomic instability and loss of imprinting is not completely understood, although multiple lines of evidence implicate the profound DNA demethylation caused by the 2i-based cocktails. During pre-implantation embryonic stages, genomic hypomethylation is transient and genomic imprinting loci remain protected [41]; however, in contrast, DNA hypomethylation in 2i-naïve hPSCs is not transient and also does not replicate the embryonic demethylation patterns [15]. The role of MEK in DNA methylation could be mediated by its stabilizing effect on UHFR1, a key factor for the recruitment of the maintenance DNA methyltransferase DNMT1 necessary for genomic stability and maintenance of imprinting [17,18,21,42,43,44].

In contrast to the above, CDK8/19 inhibition does not affect MEK activity, preserves normal UHFR1 protein levels, does not reduce global DNA methylation and does not result in karyotypic abnormalities even after long-term passage [20,21,44]. Given the loss of imprinting caused by 2i-based cocktails, here we considered of interest to determine the stability of imprinting in long-term cultured CDK8/19i-naïve hPSCs. We found that CDK8/19i-naïve hPSCs (five different cell lines, including three iPSCs and two ESCs) retain monoallelic expression and hemi-methylation of maternal and paternal imprinted genes. In the case of mouse 2i-naïve PSCs, it has been reported that female cells are more susceptible to undergo loss-of-imprinting [39,45]. In this regard, it is worth mentioning that our five tested hPSC lines include two female cell lines, WIBR3 and CB5. Moreover, teratomas generated from long-term CDK8/19i-naïve hPSCs also preserved most of their tested genetic imprints. We conclude that CDK8/19i-naïve hPSCs have a high degree of imprinting stability.

Naïve and primed hPSCs exhibit different levels of differentiation potential. Several studies have tried to model in culture the derivation of primordial germ cell-like cells (PGCLCs) from human PSCs [22,40,46,47]. One of these studies compared the ability to differentiate into PGCLCs of 2i-naïve versus primed hPSCs, observing that 2i-naïve hPSCs are more efficient in this differentiation [40]. Here, we have used the differentiation protocol reported in [22] and we have observed that briefly-treated CDK8/19i-naïve cells are as efficient as primed cells in activating PGCLC markers. Naïve cells are developmentally more distant from PGCs than primed cells, however, the differentiation protocol used does not reveal differences between the two starting PSC states. It is conceivable that the two-step PGCLC differentiation protocol, which includes the formation of EBs, minimizes possible kinetic differences. Interestingly, we have also observed that long-term cultured CDK8/19i-naïve hPSCs also retain the capacity to differentiate into PGCLCs. We conclude that the induction to CDK8/19i-naïve features in human PSCs, even after long-term culture, does not impair their capacity to generate PGCLCs.

It has been reported that 2i-naïve human PSCs present some intrinsic plasticity to express genes characteristic of the trophectoderm [48,49]. The study of human trophoblast stem cells (TSCs) has been greatly accelerated thanks to the identification of culture conditions for these cells [24]. Based on these sets of observations, it was found that briefly-treated 2i-naïve hPSCs, but not primed hPSCs, can be indeed converted into TSCs [23,50]. Given that this is a differentiation assay uniquely accessible to naïve hPSCs, but not to primed hPSCs, we wondered if briefly-treated CDK8/19i-naïve hESCs can also differentiate into TSCs. In fact, we observed with two hPSC lines that short treatment with CDK8/19i allowed TSC-differentiation. Moreover, long-term treated CDK8/19i-naïve hESCs also retained their capacity to acquire TSC identity, further corroborating the stability of the naïve state maintained by CDK8/19 inhibition.

Taken together, we conclude that CDK8/19i-naïve cells are stable after prolonged culture, preserving imprinting and potency, and capable of efficiently differentiating into embryoid bodies, teratomas, primordial germ cells and trophoblast stem cells.

## Data Availability

RNA-seq data are available from the GEO database under accession numbers GSE127186 and GSE152378. Exome-seq data are available from the NCBI (SRA) repository under accession number PRJNA629655.

## Supplementary Materials

The following are available online at https://www.mdpi.com/article/10.3390/cells10040876/s1, Table S1: primers used in this study, Table S2: RNA-seq analysis in short-term human PSCs adapted to culture in Primed or CDK8/19i conditions, Table S3: RNA-seq analysis in long-term human PSCs adapted to culture in Primed or CDK8/19i conditions, Table S4: DEG genes after short- or long-term CDK8/19i treatment.
